# Preclinical in-vivo cerebrovascular imaging in rodent models: Methods and applications in Alzheimer’s disease

**DOI:** 10.1177/0271678X261490246

**Published:** 2026-09-09

**Authors:** Diana Patalwala, Yolanda Ohene, Ben Dickie, Tim Rosenow, Isobel Thomas-Bland, Michael Nesbit, Ryu Takechi, Paul M Parizel, John Mamo, Virginie Lam

**Affiliations:** 1 Faculty of Health Sciences, Curtin Medical Research Institute, Curtin University, Perth, WA, Australia; 2 Faculty of Health Sciences, School of Population Health, Curtin University, Perth, WA, Australia; 3 Perron Institute for Neurological and Translational Research, Perth, WA, Australia; 4 National Imaging Facility, Centre for Microscopy Characterisation and Analysis, The University of Western Australia, Perth, WA, Australia; 5 Division of Psychology, Communication and Human Neuroscience, Faculty of Biology, Medicine and Health, School of Health Sciences, The University of Manchester, Manchester, UK; 6 Geoffrey Jefferson Brain Research Centre, Manchester Academic Health Science Centre, University of Manchester, Manchester, UK; 7 School of Human Sciences, The University of Western Australia, Perth, WA, Australia; 8 Department of Radiology, Royal Perth Hospital, Perth, WA, Australia; 9 Medical School, University of Western Australia, Perth, WA, Australia

**Keywords:** Cerebral blood flow, magnetic resonance imaging, neurodegeneration, preclinical imaging, ultrasound imaging

## Abstract

The cerebral vasculature maintains brain function through precise blood flow regulation and its disruption is increasingly recognised as an early and functionally significant feature of Alzheimer’s disease. Understanding these pathologies requires high-resolution imaging capturing cerebral microvascular flow in vivo, particularly in rodent models enabling longitudinal investigation. However, existing modalities involve inherent trade-offs between spatial and temporal resolution, depth, sensitivity and accessibility, while anaesthetic and methodological variability limit cross-study comparability. High-resolution optical imaging, such as two-photon microscopy, provides capillary-level visualisation but is limited by imaging depth and invasiveness. Non-invasive techniques like multi-exposure speckle imaging and optical coherence tomography assess superficial cerebral blood flow dynamics, while magnetic resonance imaging and positron emission tomography offer whole-brain coverage at lower resolution and accessibility. Contrast enhanced ultrasound presents a promising balance of resolution, sensitivity, non-invasiveness and depth penetration. Recently, ultrafast ultrasound localisation ultrasound microscopy has enabled super-resolution imaging by tracking individual microbubbles to quantitatively map microvascular flow. This review critically evaluates preclinical imaging techniques for assessing cerebral microvascular flow in rodents, highlighting their methodological considerations, strengths and limitations. We provide a framework for modality selection and identify ultrafast ultrasound localisation microscopy as the modality best positioned to deliver the standardised, longitudinal and multiscale imaging approach currently lacking.

## Introduction

Cerebral blood flow (CBF) describes the rate of arterial blood delivery to the cerebral microvasculature, expressed in millilitres per 100 g per minute.^1^ It is a fundamental physiological parameter supporting neuronal metabolism and waste removal. In the healthy brain, CBF is tightly regulated through coordinated interactions between neurons, glia, vascular cells (i.e. endothelial cells, smooth muscle cells and pericytes) and the systemic circulation. Alterations in CBF are increasingly recognised as both a contributing factor to, and a downstream consequence of Alzheimer’s disease (AD). Consequently, dysregulated CBF has emerged as a sensitive surrogate biomarker of AD,^2–4^ reflecting early cerebrovascular dysfunction.^5–7^

In humans and transgenic rodent models of AD, CBF reduction or regional redistribution often precedes neuronal dysfunction and cognitive decline, implicating vascular impairment as an early driver of pathogenesis.^8–11^ Sensitive, high-resolution imaging modalities are therefore essential to detect subtle vascular alterations in vivo, providing mechanistic insight and markers of therapeutic efficacy. Advances in preclinical imaging, including optical, magnetic resonance, positron emission tomography and ultrasound-based techniques, have improved spatial and temporal resolution, enabling quantitative assessment of microvascular flow from capillary to whole-brain scales. This review critically appraises these modalities to guide optimal selection for investigating neurovascular contributions to AD.

## Physiological parameters of cerebral microvascular flow and their relevance to Alzheimer’s disease

At the microvascular level, cerebral perfusion^12^ arises from the interplay between vessel diameter, blood flow velocity and vascular density. See Table 1 for definitions of key physiological parameters used to characterise cerebral microvascular flow. Volumetric flow through individual vessels reflects the product of blood velocity and cross-sectional area, so small changes in arteriolar or capillary diameter can substantially alter local perfusion.^13,14^ In rodent models of AD, impaired vasoreactivity,^15^ sustained vasoconstriction^16^ or pericyte-mediated capillary narrowing^17^ can produce marked hypoperfusion even without overt vascular rarefaction.^18^

**Table 1.** Definitions of key physiological parameters used to characterise cerebral microvascular flow.

Vascular physiology term	Definition
Cerebral perfusion	Volume of arterial blood delivered to a unit mass of brain tissue per unit time
Vessel diameter	Internal width of a blood vessel
Blood flow velocity	Mean speed of blood in individual vessels
Vascular density	Number and length of all the vessel segments present per unit of tissue volume
Vasoreactivity	The ability of cerebral blood vessels to constrict (vasoconstriction) or dilate (vasodilation) in response to physiological or pharmacological stimuli
Pericyte mediated capillary narrowing/constriction	Mural cells on capillary walls that contract to change capillary diameter and tone
Vascular rarefaction	Loss or reduction in the density of blood vessels within a given tissue volume
Tissue oxygenation	Amount of oxygen available within brain tissue
Capillary transit time	Time required for RBCs to traverse a capillary
Flow heterogeneity	Variability in blood flow velocity or transit time between capillaries
Transient capillary flow stall/capillary stalling	A transient interruption or marked slowing of RBC movement within a capillary
Capillary density	Number of perfused capillaries per unit tissue volume
Network architecture	Three-dimensional organisation, branching pattern and connectivity of the capillary network

Blood flow velocity further shapes tissue oxygenation by influencing capillary transit time and flow heterogeneity.^19^ Increased variability in capillary red blood cell (RBC) velocity, transient flow stalls^20^ or reduced mean velocity can impair oxygen extraction despite apparently preserved regional or global CBF, highlighting a dissociation between bulk perfusion and microvascular function.^21,22^

Capillary density and network architecture provide an additional layer of control as loss of perfused vessels reduces exchange surface area and increases diffusion distances,^23^ promoting chronic hypoxia and impaired metabolite clearance.^24^

Critically, these parameters are interdependent. A reduction in CBF may reflect upstream arteriolar dysfunction (e.g. impaired vasodilation and vasoconstriction), altered capillary flow dynamics (e.g. capillary stalling, reduced RBC velocity and pericyte-mediated constriction) or structural microvascular loss (e.g. capillary rarefaction and reduced vascular density), each with distinct mechanistic implications. Conversely, early microvascular abnormalities may be detectable at the capillary level before measurable changes in whole-brain or regional CBF occur. Preclinical imaging modalities differ fundamentally in which of these parameters they are most sensitive to, and at what spatial and temporal scales. These distinctions underpin the interpretation of modality-specific findings throughout this review, where apparent discrepancies often reflect sensitivity to different microvascular parameters rather than conflicting biological results.

Across all modalities, measured perfusion and flow metrics are additionally shaped by experimental factors such as anaesthesia choice, gas composition and physiological monitoring, which should be carefully considered when comparing studies and interpreting disease-related changes. Anaesthesia in particular is not a uniform confound. Commonly used anaesthetic agents such as isoflurane and sevoflurane are potent, dose-dependent cerebral vasodilators that increase baseline CBF and blunt neurovascular coupling relative to the awake state, which can obscure or exaggerate disease-related flow changes depending on the protocol.^25^ Cross-study comparisons of flow metrics should ideally be matched in terms of agents utilised to avoid potential confounders. See Table 2 for an overview of preclinical in vivo imaging techniques for studying cerebrovascular structure and haemodynamics in rodents, including their suitability for awake imaging.

**Table 2.** Preclinical in vivo imaging techniques to study cerebrovascular structure and haemodynamics in rodents.

Imaging technique	Depth penetration	Spatial resolution	Quantitative/derived parameters	Advantages	Limitations
2PFM/ultrafast 2PFM	~0.5–1 mm	1.3–1.5 µm	Vessel diameter^a^	Capillary-level resolution	Limited FoV
			RBC velocity^b^	Deep tissue imaging	Invasive (requires cranial window)
			RBC flux^b^	High temporal resolution (ultrafast 2PFM)	Requires fluorescent tracers
			Volumetric blood flow^b^	Most established for awake imaging	
Bessel-focus 2PLSM	~0.6–1 mm	0.5–1 µm	Vessel diameter^a^	Extended depth of field	Reduced image contrast in dense tissue
			RBC velocity^b^	Faster volumetric imaging	Invasive (requires cranial window)
			RBC flux^b^	Suitable for larger vascular networks	Requires fluorescent tracers
			Volumetric blood flow^b^		
LSI	0.3–0.5 mm	10–20 µm	Blood flow index^b^	Wide FoV	Surface imaging only
			rCBF^b^	High temporal resolution	Invasive (requires cranial window)
			Relative perfusion changes^b^	Highly suited for awake imaging	
MESI	0.3–0.5 mm	10–20 µm	Blood flow index^b^	Improved quantitative accuracy over LSI	Surface imaging only
			rCBF^b^	High temporal resolution	Complex implementation
			Relative perfusion changes^b^	Non-invasive (skull intact)	
Doppler OCT	1–2 mm	~5–10 µm	Vessel diameter^a^	Simultaneous structural and flow measurements	Limited to large vessel flow
			Axial Blood flow velocity^b^	Label-free imaging	Lower sensitivity to capillaries
			Volumetric blood flow^b^	Non-invasive	Angle dependence of velocity measurements
			Pulsatile flow velocity^b^	Increasingly being adapted for awake imaging	
Bessel beam OCM	~0.3–0.5 mm	~1–3 µm	Vessel diameter, length, tortuosity, branching density^b^	Capillary-level resolution	Limited penetration depth
			Vessel volume fraction^b^	Extended focus	Invasive (cranial window is generally required)
				Label-free imaging	
OCTA	1–2 mm	~5–10 µm	Vessel density^a^	High resolution mapping of microvascular networks	Does not directly measure blood flow velocity
			Vessel length density^a^	Label-free imaging	Restricted penetration depth
			Vessel area density^a^	Non-invasive	
				Increasingly being adapted for awake imaging	
OR-PAM	~0.5–1 mm	~1–10 µm	Vessel morphology^a^	Capillary-level resolution	Shallow depth
			Blood oxygen saturation^b^	Single-vessel imaging	Small field of view
					Often requires invasive preparation
AR-PAM	~1–5 mm	~30–100 µm	Vessel morphology^a^	Good depth-resolution balance	Slower scanning
			Blood oxygen saturation^b^	Microvascular imaging	
				Minimally invasive (scalp removal)	
OLM	~3–10 mm	~20 µm	Vessel morphology^a^	Super-resolution beyond acoustic diffraction limit	Successful imaging depends on highly absorbing circulating particles
			Blood flow directionality and velocity (with particle tracking)^b^	High cortical SNR	Computationally intensive
				Non-invasive	
OA/PAT	Whole brain (~cm)	~100–200 µm	Vessel density, tortuosity and sometimes vessel diameter (depending on resolution)^a^	Several mm to whole brain coverage	Depth-resolution trade-off
			Blood oxygen saturation^b^	3D imaging	Laser dependence
				Functional imaging	Skull attenuation
				Label-free imaging	
				Non-invasive	
Pia-flow imaging (transcranial fluorescence localisation microscopy)	~0.2–0.5 mm	~5–10 µm	Pial vessel diameter^a^	Large FoV	Limited to superficial pial vasculature
			Blood flow velocity^b^	High spatiotemporal resolution (faster than 2PFM)	Requires fluorescent microspheres
				Suitable for longitudinal imaging of pial networks	
				Non-invasive	
LMI microscopy	~0.5–1 cm	~5–10 µm	Vessel morphology, density, branching^a^	Large FoV with high capillary-level resolution	Limited penetration depth
			Relative perfusion changes^b^	Rapid acquisition; suitable for whole-cortex imaging	Requires fluorescent probes
				Non-invasive	
DCE–MRI	Whole brain (~cm)	~0.1–0.4 mm	Ktrans^a,b^	Whole brain coverage	Lower spatial resolution than optical methods
			Extravascular volume fraction^b^	Measures BBB permeability	Requires CA
			Plasma volume fraction^b^	Non-invasive	Requires compartment modelling
					Less suited for awake imaging
DSC–MRI	Whole brain (~cm)	~0.1–0.3 mm	rCBV^b^	Whole brain coverage	Lower spatial resolution than optical methods
			rCBF^b^	Measures rCBV and CBF with good sensitivity	Requires IV infusion of CA
			MTT^b^	Vascular integrity and remodelling	Less suited for awake imaging
			TTP^b^	Non-invasive	
ASL–MRI	Whole brain (~cm)	~0.1–0.3 mm	aCBF^b^	Quantitative whole brain perfusion imaging	Lower spatial resolution than optical methods
			ATT^b^	Non-invasive	Lower sensitivity to small vessels
			Labelling-related parameters		Less suited for awake imaging
BOLD–MRI	Whole brain (~cm)	~0.1–0.4 mm	Whole-brain maps of relative oxygenation/haemodynamic changes^b^	Identification and comparison of stimulus-responsive regions	Indirect measurement of blood flow changes driven by neuronal demand
			Cerebrovascular reactivity^b^	Non-invasive	Technically demanding for awake imaging
			Model-derived perfusion estimates^b^		
IVIM–MRI	Whole brain (~cm)	~0.15–0.5 mm	Pure diffusion coefficient^b^	Provides diffusion and perfusion information without CA	Lower sensitivity compared to ASL– and DSC–MRI
			Perfusion fraction^b^	Non-invasive	
			Pseudo-diffusion coefficient^b^		
[^15^O]-labelled water ([^15^O]H_2_O)-PET	Whole brain (~cm)	~0.5–1.5 mm	aCBF^b^	Deep penetration	Lower spatial resolution than optical methods
			Distribution volume	Real-time	Exposure to radioactivity
			Vascular transit time	Higher sensitivity	Requires on-site cyclotron and radiochemistry
				Non-invasive	Limited uptake in awake imaging
CE-HFUS imaging	Whole brain (~cm)	~30–75 µm	rCBV^b^	Deep penetration	Lower spatial resolution than optical methods
			rCBF^b^	Real-time	Requires CA (microbubbles)
			MTT^b^	Good sensitivity	
			Perfusion index^b^	Minimally invasive	
uULM	Whole brain (~cm)	~2.5–20 µm	Vessel density, tortuosity, diameter^a^	Super-resolution imaging	Lower spatial resolution than optical methods
			Voxel-wise blood velocity, directionality, flux^b^	Deep sensitivity to microvascular flow	Requires CA (microbubbles)
				Minimally invasive (may require craniotomy depending on the study)	Long acquisitions
				Emerging and promising modality for awake imaging	Complex processing
fULM	Whole brain (~cm)	~5–10 µm	Functional vascular maps^a^	Brain-wide microscopic functional imaging	Requires CA (microbubbles)
			Blood Velocity^b^	Minimally invasive	Long acquisitions
			Flow changes during activation^b^		Complex processing
			Neurovascular coupling metrics^b^		

FoV: field of view; RBC: red blood cell; rCBF: relative cerebral blood flow; MRI: magnetic resonance imaging; SNR: signal-to-noise ratio; Ktrans: volume transfer constant; BBB: blood–brain barrier; CA: contrast agent; rCBV: relative cerebral blood volume; MTT: mean transit time; TTP: time to peak; IV: intravenous; aCBF: absolute cerebral blood flow; ATT: arterial transit time; 2PFM: two-photon fluorescence microscopy; 2PLSM: two-photon laser scanning microscopy; LSI: laser-speckle imaging; MESI: multi-exposure speckle imaging; OCT: optical coherence tomography; OCM: optical coherence microscopy; OCTA: optical coherence tomography angiography; OR-PAM: optical resolution-photoacoustic microscopy; AR-PAM: acoustic resolution-photoacoustic microscopy; OLM: optoacoustic localisation microscopy; OA: optoacoustic; PAT: photoacoustic tomography; LMI: large-field multifocal illumination; DCE: dynamic contrast-enhanced; DSC: dynamic susceptibility contrast; ASL: arterial spin labelling; BOLD: blood oxygen level dependent; IVIM: intravoxel incoherent motion; uULM: ultrafast ultrasound localisation microscopy; fULM: functional ultrasound localisation microscopy; CE-HFUS: contrast-enhanced high frequency ultrasound.

Structural parameters are denoted by a superscript letter (a) and haemodynamic parameters are indicated by a superscript letter (b).

## Preclinical imaging techniques for detecting cerebral blood flow changes

Building on the physiological framework outlined above, interrogation of blood flow in vivo requires imaging techniques that provide clear, detailed images and quantifiable metrics, capturing both the spatial and temporal dynamics of blood movement. In rodent models of AD, a spectrum of modalities spanning microscopic (two photon microscopy), mesoscopic (laser speckle imaging, multi exposure speckle imaging, Doppler optical coherence tomograhy, optical coherence tomography angiogram) and macroscopic (dynamic contrast enhancement/dynamic susceptibility contrast/arterial spin labelling/blood oxygen level dependent/intravoxel incoherent motion MRI, [^15^O]-labelled water PET, contrast enhanced-high frequency ultrasound and ultrafast ultrasound localisation microscopy) scales have been employed.^26–29^

Collectively, these techniques quantify key physiological parameters of cerebral perfusion and microvascular function, including CBF,^30^ CBV,^31^ blood velocity,^32^ vessel diameter^33^ and arterial transit time (ATT) or mean transit time (MTT).^34^ Together, these measures describe how blood is distributed and transported through the cerebral microvasculature.

The cerebrovascular network dynamically adjusts vessel diameters to regulate local CBF and match metabolic demand. Central to these regulatory processes is the neurovascular unit (NVU), an ensemble of endothelial cells, mural cells (pericytes/smooth muscle), glial cells and neurons that orchestrates CBF responses and preserves BBB integrity. AD disrupts this system, leading to defective CBF regulation, BBB dysfunction, neurovascular uncoupling and progressive breakdown of cerebral homeostasis, which pre-clinical imaging aims to characterise in vivo.^2^

### Two-photon fluorescence microscopy (2PFM)

Among high-resolution approaches for studying CBF at the microscopic scale, 2PFM is a well-established, sophisticated intravital imaging technique that enables direct quantification of microvascular physiology in vivo. The brain cortex is typically accessed either through a thinned skull preparation or an open cranial window. Fluorescent dyes are administered intravenously, often minutes before imaging, to label the plasma blood, causing RBCs to appear as unlabelled dark objects moving within fluorescent vessels. 2PFM uses two photons of lower energy (infrared wavelength of 750–1000 nm) that are absorbed by a fluorescent molecule/dye, exciting it to a higher energy state^35^ (refer to Figure 1). The use of infrared light is less damaging to living cells and tissues compared to the shorter-wavelength visible light used in confocal microscopy.^36^ Physiologically, 2PFM imaging enables quantitative measurement of capillary flow velocity, vessel diameter changes and local oxygen partial pressure, offering a direct window into NVC and microcirculatory regulation.^37^ However, while 2PFM offers detailed insight into microvascular haemodynamics, it does not directly provide absolute CBF measurements. In AD murine models, 2PFM has revealed capillary-level pathology including transient capillary stalls caused by adhered leukocytes (affecting ~2% of capillaries), which significantly contribute to cerebral hypoperfusion and cognitive decline.^38^ Studies in TgF344-AD rats accompanied with physiological challenges demonstrated that 2PFM can quantify NVC deficits, showing abolished hypercapnia-induced vasodilatation in cortical penetrating arterioles.^39^

**Figure 1.**
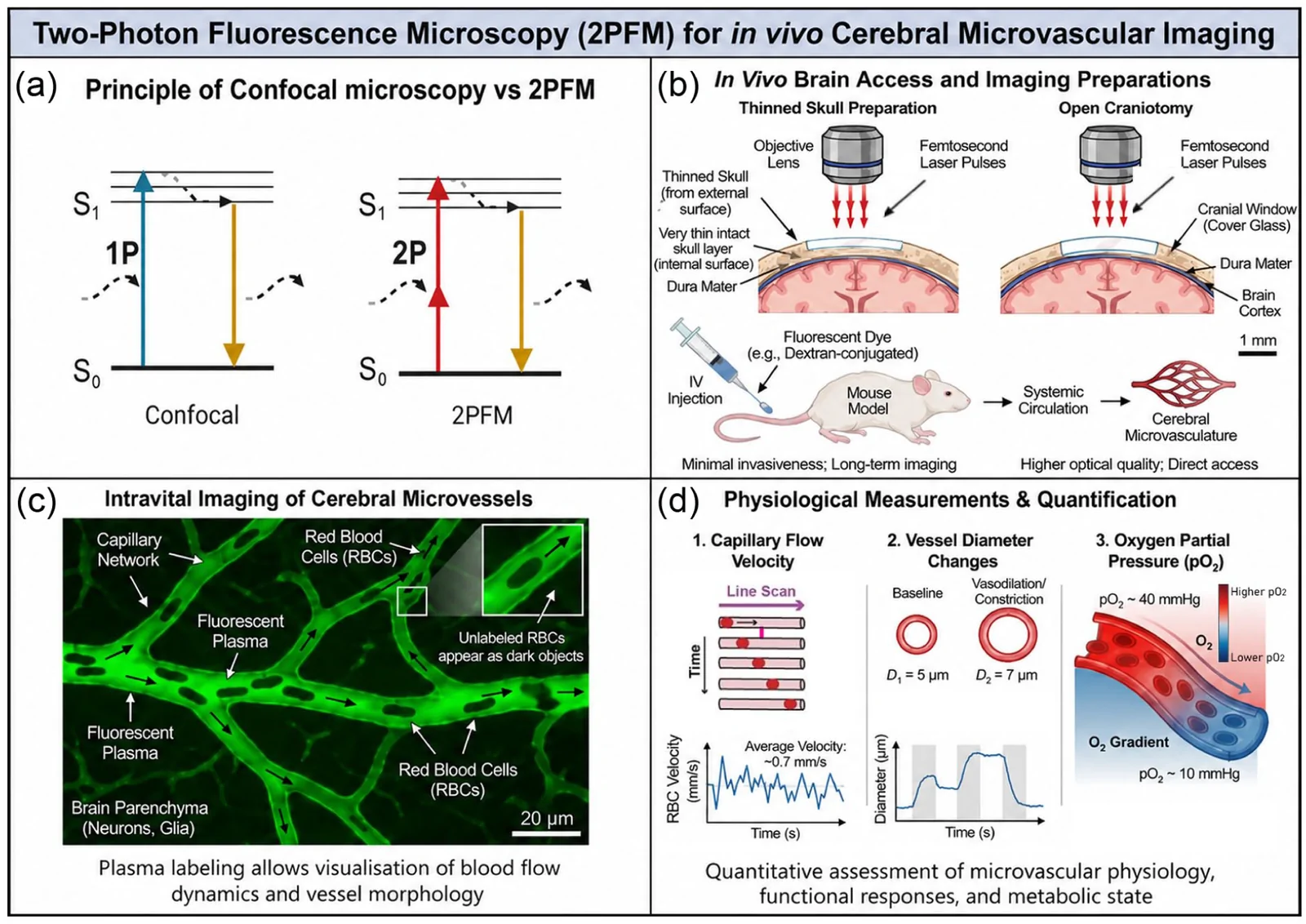
Overview of in vivo rodent 2PFM imaging: (a) comparison between principles of confocal microscopy versus 2PFM, (b) illustration of in vivo brain access and imaging preparations, (c) intravital visualisation of cerebral micro-vessels using fluorescent plasma labelling and (d) physiological measurements and quantification metrics utilised in 2PFM. *Source:* Created in SciDraw. 2PFT: two-photon fluorescence microscopy; D: diameter; IV: intravenous; pO_2_: oxygen partial pressure; RBC: red blood cell; P: photon; *S*_0_: ground state; *S*_1_: first-excited state.

Often considered the gold standard for haemodynamic imaging in terms of spatial resolution, 2PFM has been further advanced through the development of ultrafast 2PFM techniques. Ultrafast 2PFM uses rapid line-scanning strategies across vessels to track individual RBCs as they traverse vessels, enabling flow velocity quantification through spatiotemporal slope analysis (reported up to ~49 mm/s). Meng et al. demonstrated that this approach also permits calculation of RBC flux (the number of cells passing per second) in penetrating vessels. Imaging depths of up to 800 µm allows detailed assessment of the capillary network within the cortical layer.^40^

While ultrafast 2PFM is an advanced approach for maximising temporal resolution and measuring ultrafast RBC flow and flux, Bessel-focus two-photon laser scanning microscopy (2PLSM) is emerging as a complementary technique for high-speed, large-volume 3D imaging and network-scale haemodynamic measurements by using an axially elongated excitation focus to transform rapid 2D frame scans into volumetric information.^41^

Despite its strengths, a major limitation of 2PFM is that it is largely restricted to cortical regions,^42^ which restricts its ability to interrogate deep brain structures and whole-brain vascular connectivity. Other challenges include photobleaching and phototoxicity,^43^ as well as the instability of genetically encoded indicators (to record activity from defined neuronal or glial populations in cortex), which limits the ability to perform stable, repeated measurements over extended periods (e.g. several months). Cranial window surgery to gain optical access to the brain, often induces gliosis and inflammation, which may persist for days post-surgery. This inflammatory response can affect vascular physiology and confound measurements if imaging is performed immediately post-surgery, necessitating careful consideration to capture accurate physiological signals.^44^

Building on the advances established by 2PFM, a growing body of recent work demonstrates that 3PFM enables exploration of the brain at substantially greater depths. By using longer excitation wavelengths (~1300–1700 nm) and higher‑order nonlinear excitation, 3PFM enables cellular‑resolution access to deep cortical layers (>1 mm). The technique enables high-resolution fluorescence imaging of labelled blood plasma or vessel walls deep in the brain or spinal cord (e.g. cortex, white matter and hippocampus).^45,46^

In the context of neurodegeneration, 3PFM has been used to visualise fine neuronal structures and astrocytes in deep cortical layers and corpus callosum, and to map cerebrovascular morphology and haemodynamics down to hippocampal capillaries; providing tools to study microvascular changes implicated in disease models.^47^ However, for AD specifically, current work is largely methodological or proof-of-principle. While 3PFM greatly extends imaging depth, it comes with important technical constraints, such as demanding laser requirements, weak signals, heating and damage thresholds, constrained speed/field of view (FoV) and system complexity, which together currently restrict routine, large‑scale in vivo use.^48^

### Widefield optical imaging

Widefield optical imaging techniques expand the FoV to range from a few to several millimetres of the rodent brain, enabling simultaneous comparison of multiple regions and vascular compartments. These approaches utilise four distinct sources of contrast to measure various haemodynamic parameters: (1) speckle contrast, (2) optical scattering, (3) optical absorption and (4) fluorescence emission.

#### Laser speckle imaging (LSI)

LSI has been extensively applied in AD studies. In 3xTg-AD and humanised Aβ knock-in mouse models, LSI has been used to quantify resting CBF and NVC, revealing increased resting CBF (especially in females), smaller vessel diameters and impaired neurovascular responses, linking speckle-based measures to AD-relevant cerebrovascular pathology.^49^ Tarantini et al., demonstrated impaired NVC responses in TG2576 mouse model of AD using LSI.^50^

This technique works by illuminating a sample with coherent laser light, creating a random interference pattern known as a ‘speckle’ pattern on the camera sensor. When freely moving particles, such as erythrocytes, pass through this laser beam, they cause the speckle pattern to change over time. These changes in the speckle pattern are then analysed to determine the movement of the particles. The variations in the speckle pattern over time are used to infer the flow of blood within vessels.

At a functional level, LSI captures changes in speckle fluctuations, providing a sensitive indicator of relative flow dynamics in superficial cortical vessels under baseline conditions and during experimentally induced conditions such as functional activation or vascular occlusion. However, it does not directly quantify absolute flow or volumetric perfusion and is primarily limited to superficial layers of the cerebral cortex.^51^

Studies have directly compared LSI and 2PFM in the mouse cortex, showing that LSI provides robust measurements of relative surface blood flow changes, while 2PFM offers superior spatial and temporal resolution, and direct individual vessel measurements of RBC velocity.^26^ Traditional LSI is used in conjunction with a craniotomy to avoid the static scattering effects of the skull.^52^ Multi-exposure speckle imaging (MESI)^53^ can overcome this limitation by acquiring speckle images at multiple camera exposure times instead of just one.^53^ Each exposure duration samples different components of motion. Short exposures capture rapid speckle fluctuations from fast-moving blood, while long exposures integrate more of the static scattering signal (e.g. from skull or stationary tissue). By modelling how the speckle contrast changes as a function of exposure time, MESI separates static and dynamic scattering components and thus estimate a more robust blood flow index. Despite these advantages, MESI has not been widely adopted due to its complex implementation and sensitivity to model assumptions. Further investigation into the mathematical models used to describe laser speckle fluctuations reveals that the models used to calculate blood flow values vary significantly across different FoVs and vessel sizes, which drastically affects CBF quantification. Incorrect model usage can lead to errors in flow estimation, in some cases up to 300%.^54,55^

#### Optical coherence tomography (OCT)

OCT is a label-free imaging modality that generates high-resolution structural images of biological tissues based on back-reflected light. OCT uses near-infrared light (800–1350 nm) and does not rely on the use of external dyes or fluorescent labels.^56^ OCT leverages the natural properties of light to split light from a broadband, low-coherence light source into two paths: a sample arm that illuminates the area of interest and a reference arm with a movable mirror. The light reflected from both arms is recombined, forming an interference pattern. Only light travelling the same distance in both arms interferes constructively, allowing the system to measure tissue reflectivity at various depths.

With a spatial resolution of ~5–10 μm and penetration depths in millimetres, OCT provides microstructural imaging, that is, often achievable through thinned or intact skull preparations.^57^ As such, OCT occupies an intermediate position between capillary‑level 2PFM and whole‑brain modalities such as MRI or functional ultrasound.^58^

In the context of AD, OCT has been most extensively applied to retinal imaging, with recent systematic reviews summarising 14 studies across six transgenic AD mouse lines (including 3xTg-AD, APP/PS1 and 5xFAD) and detailing device configurations, retinal regions, segmentation schemes and analysis pipelines. Although limited by substantial methodological heterogeneity, these studies collectively underscore that OCT is valuable for longitudinal, in vivo assessment of retinal neurodegeneration.^59^ Nevertheless, certain intrinsic optical limitations need to be addressed. The strong scattering of light in biological tissues limits the penetration depth of OCT, restricting its ability to visualise structures located deeper within the brain. Furthermore, OCT is highly sensitive to motion artefacts caused by animal movement or pulsatile blood flow, which can reduce image quality and complicate quantitative flow analysis.^57^

#### Doppler OCT and optical coherence tomography angiography (OCTA)

Several extended functionalities of OCT have been developed to enhance its sensitivity to blood flow, acquisition speed and volumetric resolution. These include Doppler OCT and optical coherence tomography angiography (OCTA).

Both Doppler OCT and OCTA utilise phase information, more specifically changes in the phase of light waves, but they differ in their analytical objectives. Doppler OCT uses Doppler phase shifts to quantify blood flow velocity in larger vessels,^60^ and overall retinal blood flow.^61^ While it is effective for measuring blood velocity in larger vessels, it is less sensitive for imaging small blood vessels, particularly those aligned perpendicularly to the OCT beam. More recently, Bessel beam optical coherence microscopy with an extended focus has enabled imaging of the entire cortical vascular hierarchy within a ~1000 × 1000 × 360 µm^3^ field of view, while deep learning-based segmentation and integration with high-sensitivity Doppler OCT has facilitated detailed morpho-functional analyses, including three-dimensional (3-D) vascular architecture, axial and transverse blood velocity measurements, vessel-specific flow direction and graph-based assessments of vascular connectivity from individual capillaries to larger network interactions.^62^

In contradistinction, OCTA focusses on distinguishing moving particles from static tissue, generating high-resolution micro-angiograms without exogenous contrast agent.^63^ OCTA has been previously used to non-invasively detect retinal thinning, which may serve as an early biomarker for neurovascular complications in type 2 diabetes.^64^ It has also been used to longitudinally track vascular changes in large pial vessels to capillaries over a 7-month period in a mouse model of AD. A recent study by Xiang et al., showcases a reduction in superficial vessel artefacts in OCTA imaging, leading to improved visualisation and analysis of deep cortical microvasculature in an AD mouse model.^65^

These capabilities have been exploited to derive total cortical CBF consistent with autoradiographic benchmarks, to characterise capillary RBC flux and velocity and to generate 3-D CBF velocity maps that resolve arteriolar, venular and capillary responses to sensory stimuli or pharmacological challenges in both anaesthetised and awake mice.^66^

Validation studies demonstrate good correlation between OCTA-derived vessel density metrics and histological markers of perfusion, thus supporting its biological relevance.^67^ Methodologically, OCT has evolved from being a relatively invasive craniotomy-based imaging technique toward thinned-skull, intact-skull and chronic window preparations suitable for longitudinal monitoring in neurodegenerative models. High-speed swept-source systems at 1.3–1.7 µm now support large-volume, deep angiography down to the hippocampal vasculature, while enabling high-fidelity 3-D CBF velocity imaging in awake, head-fixed rodents, revealing capillary-level flow heterogeneity across cortical layers.^68^

Within a CBF imaging framework, OCT is best positioned as a mesoscopic bridge modality, relatively non‑invasive, label‑free, volumetric and quantitative, that is, well suited for cortical network‑level haemodynamic mapping and longitudinal studies. The method can both validate and contextualise perfusion MRI and ultrasound metrics while being cross‑calibrated against two‑photon measurements at selected vascular sites.

#### Optoacoustic/photoacoustic imaging

Optoacoustic (OA) or photoacoustic (PA) imaging uses nanosecond laser pulses to excite endogenous chromophores (e.g. haemoglobin), generating ultrasound waves through thermoelastic expansion. Detected acoustic signals are reconstructed into images where contrast reflects optical absorption rather than tissue mechanics. Multispectral imaging with spectral unmixing enables quantification of blood oxygen saturation (sO_2_) from oxy- and deoxyhaemoglobin signals.^69^

PA imaging includes photoacoustic tomography (PAT), photoacoustic microscopy (PAM) and related variants. PAT uses wide-field illumination and tomographic reconstruction to generate deep, three-dimensional images, and has been used to visualise mouse cerebrovascular anatomy,^70,71^ haemodynamic responses to whisker stimulation^72^ and transcranial pulse stimulation-induced changes in haemoglobin, oxygenation and vascular reactivity across the cortex of healthy (CD1) and AD (5xFAD and 3xTg) mice.^73^

In contrast, PAM acquires signals by scanning either an ultrasound detector (acoustic-resolution PAM (AR-PAM)) or a focussed laser beam (optical-resolution PAM (OR-PAM)), providing higher spatial resolution. AR-PAM has characterised microvascular morphology in SAMP8 mice,^74^ while enables microvascular imaging but generally requires scalp removal. Ultrafast functional PAM (UFF-PAM) further enables real-time, whole-brain mapping of cerebral haemodynamics and sO_2_ during hypoxia, stroke and spreading depolarisation.^75^ Optoacoustic localisation microscopy (OLM) extends PA imaging beyond the acoustic diffraction limit, achieving ~20 μm resolution at >3 mm transcranial depth while quantifying vascular structure and haemodynamics.^76^

#### Other wide field optical imaging techniques –fluorescence based

Fluorescence-based widefield optical imaging is emerging as a complementary approach for cerebrovascular imaging. Pia-FLOW uses fluorescent microsphere localisation microscopy to trans-cranially map pial blood-flow velocity, direction and vessel diameter across the intact mouse cortex, providing a faster, less invasive alternative to 2PFM for longitudinal studies in AD.^77^

Large-field multifocal illumination (LMI) fluorescence microscopy enables transcranial, cortex-wide imaging of cerebral microcirculation and perfusion at capillary-level resolution and has demonstrated longitudinal imaging of amyloid-β pathology in AD models.^78,79^

Other mesoscale fluorescence techniques, including 3-D widefield fluorescence microscopy and RBC-labelled localisation microscopy, enable volumetric vascular mapping and direct measurements of microvascular flow dynamics.^80,81^

### Magnetic resonance imaging (MRI)

MRI provides non‑invasive, whole‑brain mapping of cerebral perfusion and vascular function without cranial access. By combining a strong main magnetic field, magnetic gradients and radiofrequency pulses, MRI generates multiparametric information that reflect tissue composition, blood volume, blood flow and haemodynamic changes. When exposed to a strong magnetic field, protons in tissues align parallel or antiparallel with the magnetic field. A radiofrequency (RF) pulse is applied to temporarily change the ratio of parallel to antiparallel protons, thereby tipping the resultant net magnetisation vector by a specific angle. For example, a 90° RF pulse makes the population ratio nearly equal, whereas a 180° RF pulse inverts the population, making the antiparallel state more populated than the parallel state. Following RF excitation, the precessing protons create a changing magnetic field that, according to Faraday’s Law of Induction, induces a detectable voltage/current in a receiving coil, placed near the subject’s body. This current is the measured MRI signal. MRI contrast arises from variations in the relaxation properties (e.g. T1 and T2 relaxation times, proton density, magnetic susceptibility) of protons in different tissues.^82^ In preclinical neurodegeneration research, MRI is uniquely positioned to bridge regional vascular physiology with longitudinal disease progression, based on its deep penetration, repeatability and translational relevance to human imaging.

MRI provides whole-brain coverage and enables assessment of key perfusion parameters such as CBF, CBV and vascular MTT. Several quantitative MRI techniques can assess these perfusion parameters by exploiting distinct contrast mechanisms which render them sensitive to different components of microvascular physiology, as outlined in Table 3.

**Table 3.** Preclinical MRI techniques to assess perfusion parameters.

MRI technique	Sensitivity	Contrast mechanism	Applications
DSC–MRI	Sensitive to CBV and CBF	T2* signal changes induced by CA passage through microcirculation	Quantification of CBF and CBV
DCE–MRI	Sensitive to BBB permeability changes and CBF	Signal enhancement from T1 shortening due to GBCAs	Assessment of blood plasma volume, BBB integrity
ASL–MRI	Sensitive to CBF and ATT	Magnetically labelled arterial blood water as endogenous tracer	Non-invasive perfusion imaging, CBF quantification without CAs
BOLD–MRI	High sensitivity to blood volume and blood oxygenation changes	T2* signal changes due to paramagnetic properties of deoxyhaemoglobin	fMRI, brain activity mapping, cerebrovascular reactivity assessment
IVIM–MRI	Sensitive to microcirculatory flow	Signal attenuation upon diffusion encoding due to intravoxel incoherent motion of microvascular flow	Perfusion quantification, oncology, vascular contributions to fMRI

CBV: cerebral blood volume; CBF: cerebral blood flow; T2*: transverse relaxation time; CA: contrast agent; GBCAs: gadolinium-based contrast agents; BBB: blood–brain barrier; ATT: arterial transit time; fMRI: functional MRI; DSC: dynamic susceptibility contrast; DCE: dynamic contrast-enhanced; ASL: arterial spin labelling; BOLD: blood oxygen level dependent; IVIM: intravoxel incoherent motion.

#### Dynamic susceptibility contrast (DSC)–MRI

DSC–MRI is primarily used to quantify changes in cerebral perfusion and CBV over time. DSC–MRI focusses on the ‘first-pass’ kinetics, when a bolus of a gadolinium-based contrast agent (GBCA) rapidly passes through the capillary bed, while it remains compartmentalised within the vasculature. As the GBCA transits through the vasculature, it induces local magnetic field inhomogeneities, arising from the differences between the intra and extravascular compartments, creating mesoscopic magnetic field gradients that reduce T2* signal intensities^83^ (see Figure 2). From these signal dynamics, key haemodynamic parameters such as relative CBF and CBV can be derived.

**Figure 2.**
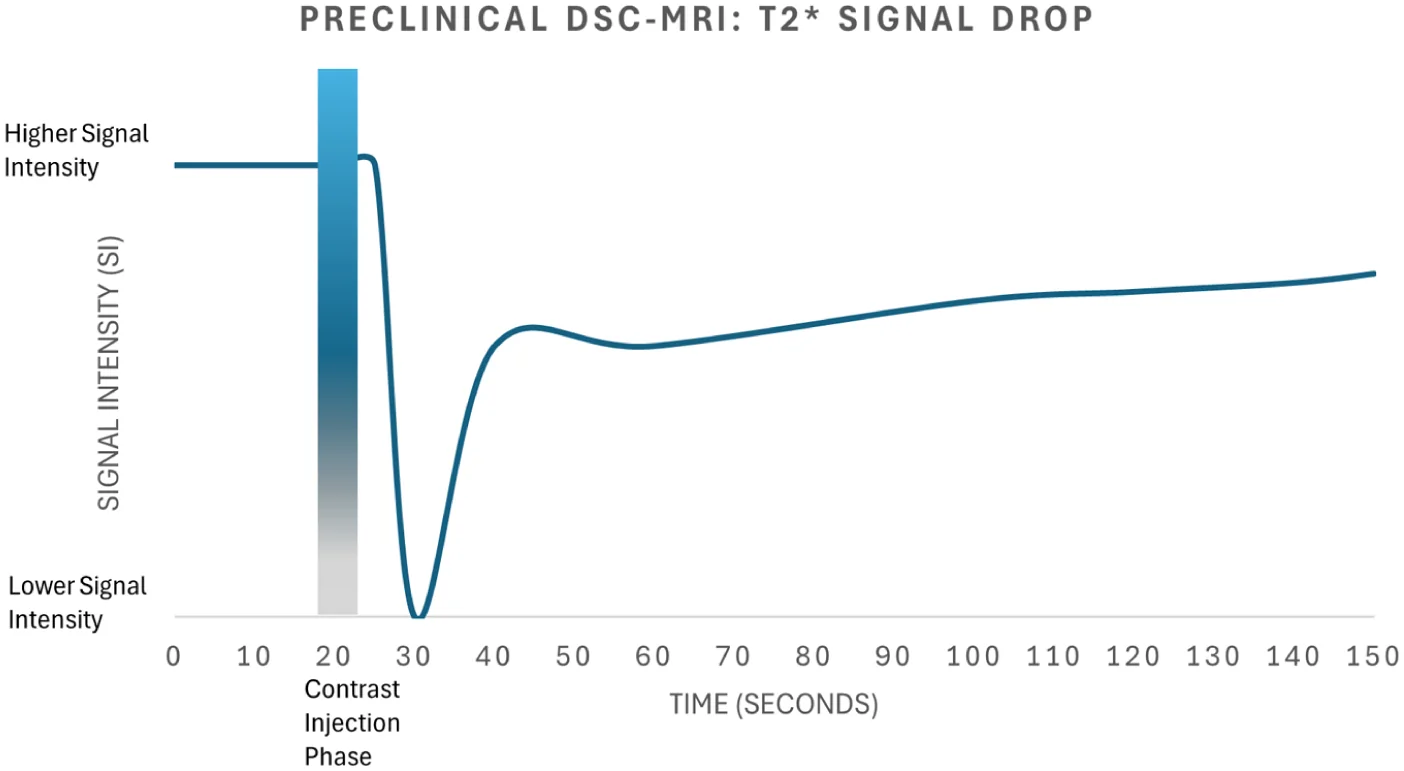
Representative preclinical DSC–MRI kinetic curve showing the characteristic first-pass T2* signal drop, recirculation and subsequent wash-out phase following contrast agent injection in a rodent model.

In transgenic AD rodent models, DSC–MRI has been used to track regional perfusion patterns change with age and pathology, and to relate vascular changes to histopathology and behavioural deficits.^84^ However, preclinical DSC–MRI is technically challenging because the rapid circulation times of small animals require optimised contrast injection protocols, accurate bolus timing and high temporal resolution.^29^ Reliable perfusion measurements depend on rapid intravenous bolus delivery using short injection lines with minimal dead volume (<10 μL), with injection rates of 5–10 μL/s reported for 0.1 mmol/kg GBCAs.^85^ High-relaxivity contrast agents (e.g. Elucirem™) may further improve bolus delivery, as their increased relaxivity allows equivalent signal effects to be achieved with smaller injected volumes, facilitating tighter bolus profiles. Capturing the first-pass bolus also requires high temporal resolution to avoid under-sampling and inaccurate perfusion estimates.^86^

Blood–brain barrier disruption presents an additional challenge, as GBCA leakage into the interstitial space can underestimate relative cerebral blood volume (rCBV), necessitating leakage-correction methods such as the Boxerman–Schmainda–Weisskoff model, although these require adaptation for rodent imaging.^87,88^ The standardisation of DSC–MRI-derived perfusion metrics also depends heavily on post-processing methodologies, and multiple initiatives have focussed on optimising and standardising acquisition protocols and analytical pipelines to improve reproducibility.^89^

#### Dynamic contrast-enhanced (DCE)–MRI

While DSC–MRI measures the T2* signal decrease (darkening) caused by intravascular GBCA during its passage through the brain, DCE–MRI measures T1 signal increase (brightening) due to GBCA leakage into the extravascular space. DSC–MRI assumes the contrast remains intravascular, while DCE–MRI relies on the contrast leakage from the intravascular to the extravascular compartment.

DCE–MRI is most valuable in AD rodent models when the primary question concerns blood–brain barrier integrity or subtle neurovascular dysfunction, particularly at early disease stages or in response to therapeutic intervention. DCE–MRI tracks the temporal first-pass kinetics of intravenously injected GBCAs, as they leak from the vascular compartment into the tissue’s extravascular space. These agents shorten the T1 relaxation time of nearby protons, resulting in signal enhancement in T1-weighted MRI images (Figure 3). Rapid, serial T1-weighted images are acquired over time to generate a signal enhancement time curve, which reflects the GBCA passing through the vasculature and leaking into tissue, enabling measurement of blood plasma volume and capillary permeability across the vascular endothelium. If the temporal resolution is high enough, CBF can also be measured.

**Figure 3.**
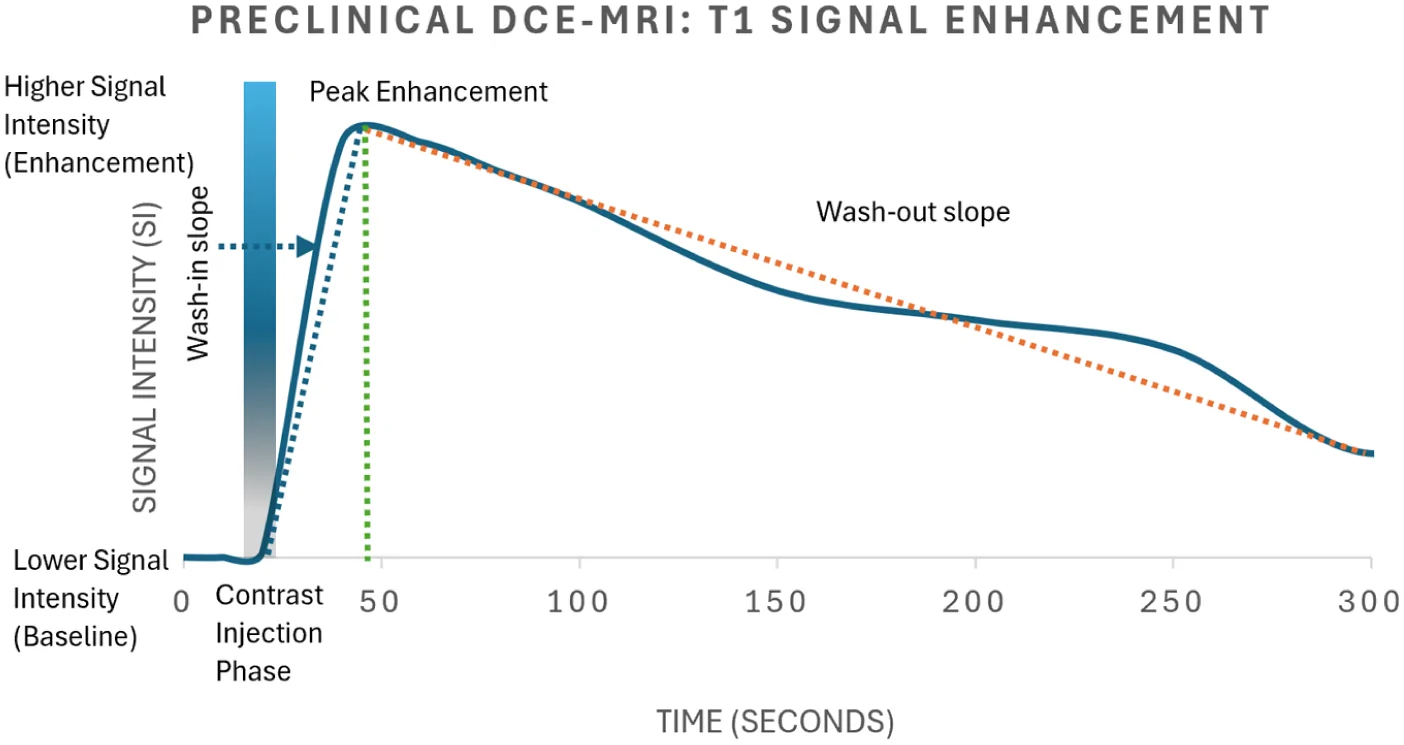
Representative preclinical DCE–MRI curve illustrating the characteristic T1 signal enhancement kinetics in a rodent model, tracking a rapid wash-in phase to peak enhancement followed by a gradual wash-out slope over 300 s.

Signal enhancement time curves can be quantified using model-free metrics such as time to peak, area under the curve and peak enhancement,^90^ although these are sensitive to acquisition parameters. To improve the accuracy and physiological relevance of the measurements, quantitative DCE–MRI converts signal changes into GBCA concentration curves and employs pharmacokinetic modelling using the arterial input function (AIF); how much GBCA is in the blood over time after injection, to estimate physiologically meaningful parameters including volume transfer constant (Ktrans), relative blood plasma volume fraction (Vp) and the extravascular extracellular volume fraction (Ve), which quantifies the relative volume of CA leakage from the microvasculature.^91^ Consensus recommendations for these parameters have been published by Dickie et al.^92,93^

DCE–MRI has been applied across AD rodent models to interrogate BBB, vascular and glymphatic function. BBB permeability, which reflects the degree to which molecules can cross the specialised brain capillary wall that normally maintains brain homeostasis,^94^ is progressively disrupted in amyloid‑driven 5xFAD mice, with 3-D ultra-short echo time golden-angle radial sparse parallel-processed DCE–MRI detecting subtle changes that appear prior to overt neurodegeneration that worsen with age relative to wild‑type controls.^95^

Despite its strengths, DCE–MRI is sensitive to acquisition parameters, modelling assumptions and signal‑to‑noise limitations, particularly in small animal brains with rapid haemodynamics.^96,97^ Careful optimisation of temporal resolution, spatial coverage and GBCA dosing is therefore essential. Although GBCAs are generally well tolerated at standard preclinical doses (0.1–0.3 mmol/kg, intravenous), repeated administration of older linear agents can result in gadolinium retention and organ toxicity, whereas macrocyclic agents exhibit lower tissue retention and improved safety.^98^

#### Arterial spin labelling (ASL)–MRI

ASL–MRI is a non-invasive perfusion technique that quantifies cerebral haemodynamics by measuring CBV, CBF and ATT without the need for exogenous CA.^99^ ASL relies on magnetically ‘tagging’ arterial blood water protons as an endogenous tracer and detecting the signal changes in tissue as labelled blood reaches the target region.^100^ To isolate perfusion-related signals, ASL alternates between acquiring labelled and control images, with the difference representing the inflowing arterial blood.^101^ Spatially-selective labelling approaches are pulsed ASL (PASL), continuous ASL (CASL) and pseudo-continuous ASL (pCASL), with PASL the most widely used^102^ (please see Table 4).

**Table 4.** Comparison of preclinical ASL–MRI labelling approaches.

Aspect	PASL	CASL (with dedicated RF coil)	pCASL
Labelling efficiency/SNR	Lowest of the three	High efficiency and SNR; optimal for small animal CBF	High, comparable to CASL when optimised and clearly higher than PASL
Hardware requirements	No extra coil, straightforward to implement on most preclinical scanner	Requires additional dedicated labelling coil in the neck; more complex set-up	No extra hardware; uses standard volume coil
Power deposition	Relatively benign because of short labelling pulses	With a dedicated labelling coil, power deposition markedly reduced (~10× lower vs volume-coil labelling)	Higher brain heating than CASL for the same labelling amplitude and acquisition duration when using a volume coil; needs careful optimisation of B1, duty cycle and scan time
Quantitative CBF accuracy	Good; limited by lower SNR	Very good; well suited for quantitative baseline CBF and activation studies in rodents	Very good; improved sensitivity to flow changes
Flexibility/protocol integration	Easy to combine with other sequences; useful as a ‘workhorse’ when absolute SNR demands are modest	Best for dedicated CBF experiments where extra hardware is available	High flexibility; can be slotted into multi-modal protocols on standard setups

PASL: pulsed arterial spin labelling; CASL: continuous arterial spin labelling; RF: radiofrequency; pCASL: pseudo-continuous arterial spin labelling; B1: oscillating radiofrequency field; SNR: signal-to-noise ratio; CBF: cerebral blood flow.

PASL uses a combination of non-selective and selective inversion pulses to label a thick blood volume, achieving high labelling efficiency in mice (~98%) and relative insensitivity to vascular anatomy.^103^ However, PASL typically has a shorter labelling duration compared to CASL, resulting in a lower signal-to-noise ratio (SNR).^104^ PASL in rat models can also be challenging as the labelling pulse does not typically reach to the heart, significantly reducing labelling efficiency.^105^

CASL, in contradistinction, labels blood directly in a feeding artery in the neck of the rodent (e.g. carotids) using continuous labelling over 1.8–2.0 s, yielding a higher SNR at the cost of slightly lower labelling efficiency (~85%). CASL typically using a dedicated RF coil positioned over the neck region to produce flow-driven adiabatic inversion.^106^ While this improves arterial labelling, it increases technical complexity compared with PASL.

pCASL was developed to combine the advantages of CASL and PASL by using a train of RF pulses and gradients to mimic continuous labelling without requiring a second RF coil. However, pCASL–MRI remains technically challenging in preclinical settings and is often limited to lower spatial resolution. The use of cryogenic coils has recently helped overcome some of these limitations, enabling improved resolution and more reliable perfusion mapping in rodents.^107^ Accurate pCASL also requires precise labelling plane positioning, while nearby air-filled structures can introduce susceptibility artefacts and off-resonance effects.^108^ Multiphase pCASL (MP-pCASL) addresses these challenges by acquiring data at multiple phase increments across 360° after RF labelling, allowing correction of off-resonance effects through model fitting. Although acquisition times are longer, the method yields accurate CBF measurements and has been validated against the ex vivo gold standard, autoradiography, in rodents.^105^

Multiple preclinical models show that ASL variants can detect hyper-perfusion, BBB dysfunction and altered water exchange before overt atrophy, aligning with vascular-first hypotheses of neurodegeneration.^109–111^ In tauopathy, ASL abnormalities occur in regions with low tangle burden and before volumetric loss, supporting its use as an early biomarker and pharmacodynamic readout.^110^

Another key variable in ASL is ATT, the time taken for labelled blood to travel from the labelling plane to the imaging voxel. Quantitative ASL models include ATT because the measured signal depends on when labelled blood arrives in the voxel. In rodents, ATT is much shorter than in humans (~100–500 ms vs ~1–2 s) due to higher cerebral blood flow and heart rates.^107^ ATT also varies with factors such as age, sex and cerebrovascular health. Multi-delay ASL can help account for this variability by sampling multiple post-labelling delays, but this increases scan time and reduces SNR.^112,113^

ASL in rodents is usually performed at a field strength of 7–9.4 Tesla, which provides an optimal balance between resolution and SNR.^114^ But because ASL is a subtraction-based technique relying on labelled and control images, even minor animal head movement can introduce substantial artefacts, making rigorous motion control and post-processing correction essential.

Despite these challenges, the close correspondence between rodent and human ASL models and consensus standards makes ASL a highly translational perfusion technique.

#### Blood oxygen level dependent (BOLD)–MRI

BOLD–MRI is a functional imaging technique that indirectly assesses changes in haemodynamic responses through NVC, that is, the physiological link between neuronal activity, blood flow and oxygen metabolism. Rather than measuring absolute perfusion, BOLD–MRI is particularly suited to detecting relative or stimulus-evoked haemodynamic changes.

BOLD–MRI relies on the differences in the magnetic properties of haemoglobin: oxyhaemoglobin (oxygen-carrying form) is diamagnetic, while deoxyhaemoglobin (oxygen-depleted form) is paramagnetic (Figure 4).^115^ This difference alters local magnetic field homogeneity, influencing the MRI signal, particularly in T2*-weighted images. When a brain region becomes active, neuronal activation triggers NVC, increasing local CBF and CBV. These haemodynamic changes increase oxygen delivery more than oxygen consumption, reducing the local concentration of deoxyhaemoglobin. Together, the reduction in deoxyhaemoglobin and the concomitant blood volume changes alter local magnetic susceptibility and produce the measurable increase in the BOLD signal.^116^

**Figure 4.**
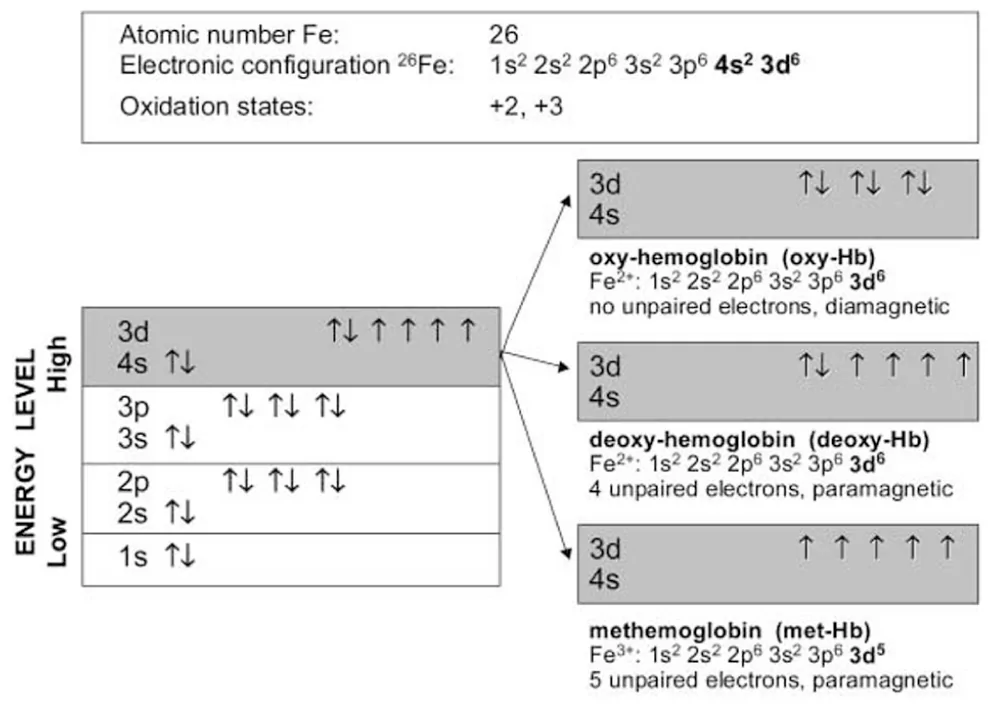
Electron distribution in the orbitals of iron (energy levels), in oxidation states +2 and +3, in various haemoglobin degradation products. *Small arrows* indicate magnetic dipole moments of single electrons. In each orbital, magnetic moments pair with those with opposite magnetic di-pole moment. In unfilled orbitals, electrons remain unpaired until the shell is half full. In oxyhaemoglobin, there are no unpaired electrons in the third orbital. The substance is therefore diamagnetic. In deoxy-haemoglobin there are four unpaired electrons in the third orbital. This substance is paramagnetic. The 4-s orbital is empty in both cases, because iron is in the ferrous (+2) state. In methemoglobin (in certain pathologies, deoxyhaemoglobin is oxidised to methemoglobin) the third orbital contains five unpaired electrons. Due to the loss of one additional electron, iron is now in the ferric (+3) state. *Source:* Reproduced with permission from Parizel et al.^115^

In AD mouse models, dynamic BOLD–MRI combined with transient hypoxic challenges has been used to generate non-invasive whole-brain maps of cerebral perfusion. In Tg‑SwDI transgenic mice (2–6 months old), a brief 5‑s hypoxic gas pulse is applied during whole‑brain BOLD–MRI to generate an endogenous deoxyhaemoglobin ‘bolus’, which acts as a paramagnetic CA. By modelling the resulting BOLD signal with a partial‑volume corrected Arterial Input Function, quantitative maps of CBF and CBV were obtained across the entire brain. Using this approach, Tg‑SwDI mice showed significantly reduced CBF and CBV preceding cerebral atrophy, indicating that perfusion deficits emerge early and may serve as a sensitive biomarker of prodromal dementia.^117^

A comprehensive review of preclinical MRI for microvasculature, highlights the quantitative BOLD (qBOLD) technique, which derives deoxygenated blood volume and oxygen extraction fraction in rodents, parameters highly relevant to microvascular integrity.^102^ Further, calibrated BOLD-functional MRI (fMRI) in mice combines CBF (ASL) with BOLD-derived relaxation to estimate cerebral metabolic rate of oxygen, providing high-resolution haemodynamic mapping that can be translated into disease models.^118^ These methods enable early detection of vascular dysfunction before overt neurodegeneration.

Despite its strengths and translational relevance, rodent BOLD–MRI is strongly influenced by experimental conditions such as anaesthesia choice, physiological state and motion/stress in awake setups which may skew signal interpretation.^119,120^ Careful protocol design, rigorous physiological monitoring and preprocessing are essential to avoid misattribution of vascular changes to neuronal activity.

#### Intravoxel incoherent motion (IVIM)–MRI

IVIM–MRI quantifies microvascular perfusion by measuring signal attenuation caused by capillary microcirculation.^121^ It is a diffusion-weighted imaging (DWI) technique that distinguishes between molecular diffusion in tissue and perfusion-related motion. IVIM does not require contrast administration. Unlike conventional DWI which typically employs b-values ranging from 300 to 1000 s/mm^2^ to quantify the apparent diffusion coefficient (ADC) of water within tissue, IVIM aims to capture rapid incoherent, motion of blood within the microvasculature by using multiple lower *b*-values (<300 s/mm^2^). Since the lower *b*-values attenuate blood while having minimal effect on tissue water, perfusion-related IVIM fraction (fIVIM) and the pseudo-diffusion coefficient (D*) can be separated from the tissue ADC.^82^

In rodent studies, IVIM parameters have demonstrated physiological relevance. In rats, regional variations in vascular density across cortex, corpus callosum, hippocampus, thalamus and hypothalamus show a significant positive association with the IVIM parameter known as D*, supporting its use as a surrogate marker of perfused vascular density in healthy rodent brain.^122^ More recently, alterations in fIVIM and D* have been linked to microvascular integrity and regional hypoperfusion in models of small‑vessel disease and neurodegeneration, suggesting sensitivity to disease‑related microcirculatory dysfunction.

Methodological refinements continue to improve IVIM specificity, including approaches that account for exchange across the BBB using crusher-compensated Filter-Exchange Imaging,^123^ and measuring velocity distributions via modelling,^124^ or flow-compensated gradients.^125^ These advances aim to better disentangle capillary flow, exchange and diffusion contributions to the IVIM signal.

Further research is required to refine IVIM models and characterise how blood flow dynamics at varying spatial and temporal scales influence IVIM signals. The commonly used mono-exponential decay model remains suitable in most cases. At higher *b*-values, non-Gaussian diffusion effects and noise impact MRI signal accuracy, while at lower *b*-values, these factors influence IVIM parameter estimation. To ensure accurate quantification, appropriate handling of the diffusion component, typically accounting for over 90% of the total signal at low *b*-values, is crucial.^121^ Further validation is required to clarify how IVIM metrics relate to specific microvascular alterations across different neurodegenerative disease models and stages.^82^

### Positron emission tomography (PET)

PET is a nuclear medicine imaging modality that quantifies the distribution of radioactively labelled tracers in vivo. By applying tracer kinetic modelling, it provides valuable insight into absolute and relative physiological and metabolic processes. Unlike optical approaches, PET offers whole brain coverage, including deep structures, and provides absolute perfusion estimates when combined with tracer kinetic modelling.

In preclinical AD research, PET is useful in quantifying regional CBF and valuable for longitudinal biomarker development, although practical constraints include infrastructure requirements, cost, radiation exposure and lower spatial resolution relative to microscopy-based techniques.

#### [^15^O]-labelled water PET ([^15^O]H_2_O PET)

Absolute quantification of CBF can be achieved using PET with intravenous administration of [^15^O]H_2_O, because water freely diffuses across the capillary membrane and kinetics can be modelled to estimate perfusion. This technique is considered the gold standard for CBF quantification when combined with arterial blood sampling to establish AIFs.^126–128^

The conventional method, blood-sampled input function, involves arterial cannulation and serial blood sampling (to measure whole-blood radioactivity concentration vs time), which despite its accuracy, presents procedural challenges, potential errors and notable risks, especially in small animal studies where blood volume constraints limit feasibility.^129–132^

As an alternative, image-derived input functions (IDIF) utilise visible blood pools within the PET FoV to extract an input curve. In neuroimaging, carotid arteries are commonly used due to their prominence within the FoV. Co-registration of PET images with high-resolution MRI enables MRI-derived carotid artery segmentation, enhancing IDIF accuracy. However, this approach requires MRI availability and additional processing pipelines, which may not always be practical. Advances in PET technology have improved standalone PET-based IDIFs by leveraging high-resolution imaging, but these systems remain limited in accessibility, necessitating alternative compensatory techniques for standard-resolution PET scanners.

Recent artificial intelligence (AI)-driven methods offer a promising avenue for estimating blood time-activity curves directly from PET images, reducing dependence on invasive arterial sampling. However, the generalisability of AI models across different animal species, perfusion states and imaging conditions remains uncertain, requiring further validation.^133^

[^15^O]H_2_O PET is widely used for quantitative regional CBF in rats, often with arterial input or arteriovenous shunts for blood sampling.^134^ The technique is sensitive to vasodilatory challenges such as acetazolamide.^135^ Alternatively, [^15^O]-labelled gases can be used to measure CBF and cerebral metabolic rate of oxygen in neonatal rats via spontaneous inhalation, eliminating the requirement for vascular cannulation.^136^ Most preclinical applications focus primarily on ischaemia, hypoxia or vascular dysfunction. With appropriate logistics including cyclotron access, small-animal PET, optimised sampling or noninvasive input-function strategies, these protocols can be adapted to AD rodent models for sensitive, longitudinal perfusion assessment.

#### [^11^C]N-isopropyl-p-methylamphetamine PET ([^11^C]MMP)

[11C]MMP is a newer PET tracer designed as a high‑resolution alternative to [^15^O]H_2_O for measuring CBF.^137^ The regional influx rate constant of [^11^C]MMP reflects regional CBF at rest and increases following acetazolamide challenge, consistent with hyper-perfusion and confirming sensitivity to CBF. At high flows after acetazolamide, [^11^C]MMP underestimates CBF in higher‑flow regions, suggesting non‑linearity at the top end of the flow range. The early-phase standardised uptake value (0–10 min) shows good correlation with regional CBF, supporting the use of short dynamic scans as a practical semi-quantitative index.^138^ Overall, [^11^C]MMP is a promising new PET tracer for CBF imaging, offering improved image resolution relative to [^15^O]H_2_O and the feasibility of shorter scan protocols. Its main limitation is underestimation at very high flows and the logistical need for ¹¹C production due to its short half-life. Presently, its application remains in the preclinical/early-translation phase and needs further validation across disease states and physiological conditions relevant to AD.

### Contrast-enhanced high-frequency ultrasound (CE-HFUS)

Many advanced imaging modalities provide excellent spatial resolution but are limited by shallow penetration depth, while whole-brain imaging techniques often lack sufficient spatial resolution. CE-HFUS bridges this gap by enabling imaging of CBF across the entire rodent brain with deeper penetration than optical methods and higher spatial and temporal resolution than many whole brain modalities.^139^

Modern preclinical ultrasound systems employ high-frequency transducers, achieving spatial resolution as fine as 30 µm per pixel.^140^ Both non-contrast and contrast-enhanced ultrasound modalities can be utilised for CBF assessment. Traditional non-contrast Doppler ultrasound effectively detects flow in large, high-velocity vessels (e.g. arteries and arterioles) but lacks sensitivity to capillary networks.^141,142^ In contrast, CE-HFUS enables visualisation of both large and small vessels, including capillaries, through the use of intravenously administered microbubble CAs, which are detectable using non-linear contrast mode imaging.

Microbubbles, typically 1–10 µm in diameter, contain high molecular weight/low-solubility gases, such as perfluorocarbons or sulphur hexafluoride, stabilised by lipid, protein or polymer shells.^143,144^ Albumin, biocompatible polymers or lipids are commonly employed to stabilise microbubbles, helping to prevent their coalescence and minimise gas dissolution in the bloodstream. Their micron-scale diameter keeps them confined to the vascular compartment, allowing for a direct and precise assessment of perfused blood volume and flow.^145^ Due to their substantial ultrasound scattering intensity over 1000 times that of erythrocytes, microbubbles significantly enhance backscattered echo signals, facilitating superior imaging contrast.^146,147^

Quantification is based on image intensity or the timing of bubble uptake, with the average intensity plotted as a time-intensity curve (TIC) to assess perfusion dynamics.^145^ Advanced mathematical models are often used to refine TICs and extract key perfusion parameters like time-to-peak, peak intensity/enhancement, wash-in-rate and the like.^148^ CE-HFUS is particularly advantageous in research settings, providing measurements of capillary blood volume and velocity.^149^

CAs can be delivered by a bolus injection (wash-in kinetics) as well as by constant infusion involving a high mechanical index destructive ultrasound pulse resulting in a replenishment curve. The advantages of destruction-replenishment model are that the transit of the CA through the cardiopulmonary system no longer influences the perfusion results, multiple imaging planes can be quantified with constant infusion and a steady-state of microbubbles, allowing flow measurements to be repeated in quick succession^150^ (refer to Figure 5).

**Figure 5.**
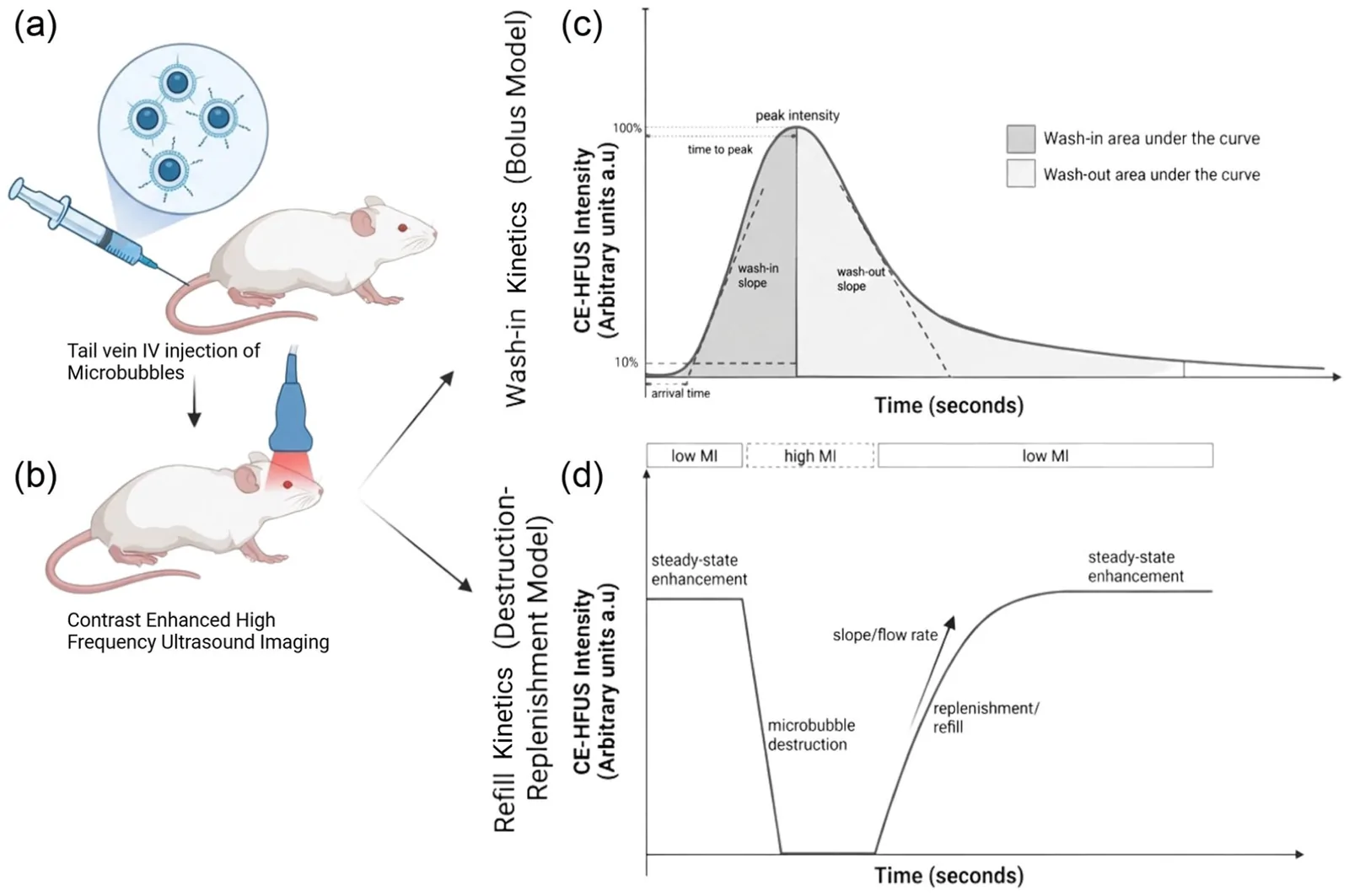
CE-HFUS workflow illustration: (a) microbubble tail-vein injection, (b) CE-HFUS imaging, (c) wash-in kinetics involving bolus injection and (d) refill kinetics involving constant infusion of microbubbles followed by a high-frequency ultrasound burst. *Source:* Created in BioRender. IV: intravenous; a.u: arbitrary units; CE-HFUS: contrast-enhanced high frequency ultrasound imaging; MI: mechanical index.

Despite its advantages, CE-HFUS remains highly operator-dependent and demands a thorough understanding of both ultrasound CAs (UCA), the imaging system, as well as detailed knowledge of animal physiology. While clinical guidelines for the use of UCAs have been established,^151^ comparable protocols for preclinical applications remain underdeveloped. A crucial consideration is the oscillatory behaviour of microbubbles, which varies with acoustic parameters such as pulse length, amplitude and frequency. Improper parameter selection can degrade image quality, prematurely destroy microbubbles or even cause tissue damage.^152,153^ Additionally, microbubbles exhibit a short circulation half-life (typically minutes) due to rapid clearance by the reticuloendothelial system.^154^ This necessitates repeated injections for extended imaging, which may influence physiological responses.^155^ Variability in microbubble size, concentration and stability can introduce inconsistencies, underscoring the importance of standardised protocols.^156^ To mitigate bubble separation and aggregation, gentle agitation of the syringe or infusion system is highly recommended during administration.^149^

CE-HFUS requires a rigorous step-by-step protocol for the preparation and use/injection of the UCA, animal handling and monitoring and determining the ultrasound acquisition and analysis settings. Standardising these procedures helps reduce the variability between operators and research centres.

#### Ultrafast ultrasound localisation microscopy (uULM)

uULM is an advanced imaging technique that combines deep tissue penetration with super-resolution imaging, offering spatial and temporal detail by capturing up to a thousand frames per second. The high frame rate allows separation of individual microbubbles because their echoes fluctuate over successive frames as they move with the blood, enabling precise tracking of individual bubbles in both space and time for accurate measurement of blood flow speeds over a large dynamical range. Since microbubble signals change much faster than tissue signals, applying a differential subtraction filter of consecutive frames, effectively removes background tissue signals and highlight the bubbles more efficiently. Because ultrasound can reach deep into tissue, this technique can potentially map even the smallest blood vessels, down to capillaries. uULM leverages ultrasound’s deep tissue penetration to map microvascular networks, including capillaries, non-invasively. Imaging can be performed through a thinned skull or even intact skull, though the latter attenuates signals and reduces resolution, limiting detection of vessels around 20 µm in diameter and up to depths exceeding 8 mm.^139^ Open-skull preparations (craniotomy) still provide the best image quality and finest microvascular detail, particularly at capillary scales.^157^ Combining sensory stimulation with ultrasound localisation microscopy, results in functional ULM (fULM). By tracking spatiotemporal changes in microbubble dynamics over repeated acquisitions, fULM has enabled the investigation of neurovascular coupling and blood flow responses associated with sensory stimulation.^33^

The main challenge in uULM is detecting enough individual microbubbles in the blood to create high-resolution maps of blood vessels quickly. In a study involving anaesthetised male Sprague–Dawley rats, an uULM system (such as the widely used Iconeus platform) automatically acquired 75,000 ultrasound images over 150 s and processed them to identify microbubbles based on how quickly their signals changed (decorrelated).^139^ These changes can happen because of movement, disruption or dissolving of the bubbles, so the sequence uses special low‑mechanical‑index settings to avoid destroying the bubbles. Advanced filtering techniques helped remove background signals from tissue and bone, allowing the bubbles to stand out clearly. The system then localises bubble positions with very high precision, down to about 2.5 μm. The technique could benefit further by utilisation of raw ultrasound data to correct for signal distortion caused by the skull.^158^

More recently, 3-D transcranial uULM in mice has enabled whole-brain vascular imaging at unprecedented vascular scale. Cerebral microvascular imaging was successfully performed across the entire brain, capturing a wide range of vessel sizes, from 20 to 200 µm, and flow speeds ranging from approximately 2 to 100 mm/s.^159^ 3-D uULM, however has prolonged processing time, although emerging deep learning approaches aim to accelerate analysis.^160^

## Conclusion

Progress in preclinical cerebral microvascular imaging depends on progress along two fronts: closing the persistent trade-off between spatial resolution and imaging depth and reducing the methodological variability that currently limits reproducibility across studies. Technological innovation alone is insufficient; rigorous standardisation of experimental design, acquisition protocols and analytical frameworks is equally necessary. Factors such as anaesthesia (agent, dose, gas composition, monitoring and reporting),^25,161–164^ contrast agents (choice, dose, timing, injection route, pharmacokinetics)^165–167^ and post-processing methodologies (kinetic models, fitting, motion correction, atlas registration and quality control),^91,168^ can profoundly influence measured haemodynamic parameters and complicate comparisons between studies. Given these agent-specific effects on baseline and stimulus-evoked flow, there is growing recognition that, where feasible, awake imaging may provide a more physiologically relevant assessment of vascular dynamics in AD and improve cross-study comparability.

Among current in vivo techniques, two-photon fluorescence microscopy is widely regarded as the most mature and informative approach for awake cerebral microvascular imaging, owing to its compatibility with chronic cranial windows,^169,170^ established head-fixation protocols^171,172^ and extensive validation in longitudinal studies.^173^ Laser speckle contrast imaging is also highly suited to awake preparations because of its rapid acquisition and relative robustness to physiological fluctuations, making it a common choice for repeated monitoring of cortical perfusion.^170^ Optical coherence tomography angiography and Doppler OCT are increasingly being adopted in awake mice and rats, offering an attractive balance between spatial coverage and vascular detail, although motion artefacts remain a technical challenge.^66,174,175^ Ultrasound localisation microscopy is emerging as a particularly promising modality for awake imaging due to its ability to interrogate deep brain structures and larger-scale vascular networks.^176^ In contrast, MRI-based approaches remain comparatively less suited to routine awake imaging because they require extensive animal habituation, specialised restraint systems and sophisticated motion-correction strategies. Similarly, techniques such as PET and photoacoustic imaging have seen more limited uptake in awake cerebrovascular studies owing to practical constraints related to acquisition duration, instrumentation and sensitivity to motion. Awake-imaging compatibility is not, however, the only criterion on which modality selection should be based on; resolution, depth and throughput matter equally, and it is here that the field’s central trade-off becomes most apparent.

This review highlights the complementary strengths and limitations of preclinical imaging modalities used to assess cerebral microvascular flow in rodent models of AD. High-resolution optical methods, such as two-photon microscopy and wide-field optical imaging approaches, provide unparallel spatial resolution, facilitating quantitative measurements of capillary flow, vessel diameter, red blood cell velocity and neurovascular coupling but are constrained by invasive cranial preparations and limited imaging depth for characterising whole-brain cerebral blood flow. Non-invasive optical modalities, including multi-exposure speckle imaging and optical coherence tomography angiography, extend capability to cortical surface imaging but remain restricted in deeper regions. Magnetic resonance imaging and positron emission tomography provide highly translational whole-brain coverage and quantitative assessment of perfusion parameters, allowing multi-parametric evaluation of cerebral blood flow, blood volume and permeability but are limited by cost, accessibility and lower spatial resolution to resolve capillary-scale abnormalities, which are increasingly being recognised as early drivers of AD. No single modality in current use therefore satisfies both the resolution and the whole-brain coverage that mechanistic and translational AD research ultimately requires.

The key unmet need, therefore, is not simply higher resolution or greater depth, but a standardised, multi-scale framework capable of linking capillary-level dysfunction to network-level perfusion changes within the same biological system over time. Such a framework would allow the arteriolar, capillary and structural mechanisms of perfusion kinetics outlined earlier in this review to be tracked longitudinally within the same animal, rather than inferred by combining separate cross-sectional studies that differ in resolution, depth and anaesthetic protocol. In our view, this gap, rather than any single modality’s resolution or depth limitation in isolation, is the principal barrier to translating preclinical microvascular findings into reliable, disease-tracking biomarkers of AD.

Among emerging approaches, ultrasound-based imaging is best positioned to meet this need. Contrast-enhanced high-frequency ultrasound and ultrafast ultrasound localisation microscopy in particular bridge the resolution-depth gap by combining deep penetration, high spatial resolution and sensitivity to microvascular dynamics. By combining the deep tissue penetration and real-time acquisition capabilities of ultrasound with microbubble-based super-resolution vascular mapping, uULM bridges the longstanding gap between microscopic optical techniques and macroscopic whole-brain imaging modalities. Recent demonstrations of transcranial whole-brain vascular imaging, quantitative flow measurements across a broad range of vessel calibres and the potential for minimally invasive longitudinal studies position uULM as a promising emerging modality for investigating neurovascular dysfunction in rodent models of AD. Although challenges remain, including acquisition complexity, computational demands and the need for further validation and greater standardisation, ongoing technological advances are rapidly addressing some of these limitations.

Therefore, while modality selection should remain guided by the specific biological question being addressed, current evidence suggests that uULM represents a potential next-generation platform for comprehensive in vivo assessment of cerebral microvascular flow in rodents. Its unique ability to combine microvascular-scale resolution with whole-brain coverage and the potential for minimally invasive and awake imaging paradigms, places it in a strong position to become an important reference technique for studying the vascular contributions to neurodegenerative diseases such as AD.
